# An analysis of anxiety and depression in second-trimester pregnant women with cervical insufficiency

**DOI:** 10.3389/fpsyt.2026.1749801

**Published:** 2026-03-27

**Authors:** Jiebing Chen, Dayue Liu, Qiqiao Du, Guimei He, Chunqi Luo, Meifong Lam, Fengchun Wu, Jinglei Ma, Yinglei Mo, Junxiu Liu

**Affiliations:** 1 Department of Obstetrics and Gynecology, The First Affiliated Hospital, Sun Yat-sen University, Guangzhou, China; 2 Medical Affairs Department, The First Affiliated Hospital, Sun Yat-sen University, Guangzhou, China; 3 Psychiatric Service of the Centro Hospitalar Conde de São Januário, Macao, Macao SAR, China; 4 Department of Psychiatry, The Affiliated Brain Hospital, Guangzhou Medical University, Guangzhou, China; 5 Department of Operative Dentistry and Endodontics, Guanghua School of Stomatology, Guangzhou, China; 6 Guangdong Provincial Key Laboratory of Stomatology, Sun Yat-sen University, Guangzhou, China; 7 Nursing Department, The First Affiliated Hospital, Sun Yat-sen University, Guangzhou, China

**Keywords:** anxiety, cervical insufficiency, depression, pregnant women, second trimester

## Abstract

**Objective:**

Perinatal mood disorders can seriously endanger the health of pregnant women and fetuses, thus causing heavy burdens and potential hazards to families and society. This study aimed to investigate anxiety and depression in second-trimester pregnant women with cervical insufficiency and provide guidance for clinical practice.

**Methods:**

A total of 136 second trimester women with cervical insufficiency who underwent laparoscopic cervical cerclage were selected as the observation group, and 117 Pregnant women with no pregnancy complications diagnosed in the second trimester of pregnancy composed the control group. In addition, both online and paper questionnaires were designed to collect basic information. Moreover, Zung’s Self-Rating Anxiety Scale and Self-Rating Depression Scale were employed to evaluate the anxiety and depression of participants in both groups.

**Results:**

Pregnant women in the cervical insufficiency group had significantly higher Self-rating Anxiety Scale and Self-rating Depression Scale scores than did those in the control group (both P<0.001). Compared with those in the control group, a greater percentage of pregnant women in the cervical insufficiency group met the diagnostic criteria for anxiety and depression. The multivariate linear regression model revealed that cervical insufficiency and a history of abnormal pregnancy were related to higher Self-rating Anxiety Scale scores, whereas a history of abnormal pregnancy was also associated with higher Self-rating Depression Scale scores (both P<0.05).

**Conclusion:**

Pregnant women with cervical insufficiency are more likely to suffer from anxiety and depression in the second trimester than normal pregnant women are. Healthcare professionals should pay more attention to these patients in clinical practice.

## 1 Introduction

Cervical insufficiency (CI) is defined as painless dilatation with shortening of the cervix in the absence of other causes before the 37th week of pregnancy. This disease may result in membrane prolapse, premature rupture of the membrane, second-trimester miscarriage, or preterm birth. As one of the underlying causes of pregnancy loss and premature birth, CI accounts for 14.3−65% of premature births (1–3). Second-trimester miscarriages often occur at identical pregnancy weeks. As the labor process progresses rapidly, adverse pregnancy outcomes are usually inevitable. Fetal loss caused by CI commonly occurs between 16 and 28 weeks of gestation (4).Preliminary investigations demonstrated that scores on the Self-rating Depression Scale (SDS) and Self-rating Anxiety Scale (SAS)—objective measures of anxiety and depressive symptoms—were significantly higher in pregnant women with cervical incompetence (CI) than in those without diagnosed pregnancy complications (5). In the second trimester, pregnant women can experience the joy of becoming a mother through a fetal heartbeat and fetal movement and foster a close relationship with their babies. Consequently, pregnancy loss at this stage can cause irreversible damage, not only physically but also, most importantly, psychologically (6, 7). Susil et al. investigated 137 women and reported that involuntary termination before 28 weeks of gestation could lead to adverse mental health consequences, with nearly one-fifth of women fulfilling the criteria for depression and over half of women fulfilling the criteria for complicated grief after spontaneous abortion (8). The increasing age, declining fecundity, and desire to own a child can all impose an enormous psychological burden. Continuous perinatal mood disorders may result in tension between couples, poor family relationships, and even suicidal tendencies (9–11).

Previous adverse pregnancy outcomes significantly affect the psychological state of pregnant women, especially during crisis periods. He and Wang et al. explored 1138 nonpregnant women and revealed that those with previous nonvoluntary miscarriages had a significantly greater incidence of anxiety and depression (12). Craig et al. conducted an analysis of 81 women with recurrent miscarriages and reported that nearly one-sixth of the patients were diagnosed with depression and that one-fifth of the women experienced chronic anxiety (13). Perinatal depression and anxiety disorders may have devastating effects on women, infants, and their families, as well as potential long-term effects on babies’ cognition, emotion, and behavior. In some serious cases, patients even ultimately commit suicide or infanticide, thus leading to catastrophic impacts on their family and society (14). Hence, identifying and preventing perinatal mood disorders in clinical practice is highly important.

Several surgical modalities have been proposed for the treatment of CI, including transvaginal and transabdominal cervical cerclage, the latter of which can be accomplished through open laparotomy or laparoscopic surgery. Since Karen B et al. reported the first case of cervical cerclage *via* laparoscopy in 1998 (15), this type of cerclage has become popular worldwide. In addition, the safety and effectiveness of this technique have been confirmed. Chen et al. reported that laparoscopic cervical cerclage can significantly prolong gestational age compared with conventional vaginal cerclage (16). However, even with the protection of cervical cerclage, these women might still suffer from considerable psychological pressure during pregnancy, especially around the crisis period, which may lead to a higher incidence of perinatal mood disorders. However, few studies have focused on the psychological status of pregnant women with CI. Therefore, it is necessary to conduct detailed analyses of the mental health of pregnant women with CI.

In this study, depression and anxiety in second-trimester pregnant women with CI and relevant influencing factors were analyzed in an attempt to provide some reference for clinical practice and nursing care.

## 2 Methods

### 2.1 Study design and participants

A total of 136 CI patients who received treatment in the gynecology department of the First Affiliated Hospital of Sun Yat-sen University in Guangzhou from April 2019 to February 2020 were enrolled in this study. The inclusion criteria were as follows: (1) pregnant women in the second trimester, (2) Patients diagnosed with cervical insufficiency by gynecologists at our hospital and who underwent laparoscopic cervical cerclage, (3) pregnant women who had never been diagnosed with mental illness or other neurological disorders previously, and (4) pregnant women who were able to read and communicate normally and who participated in this survey voluntarily. The exclusion criteria were as follows: (1) Pregnant women with a psychiatrist-diagnosed psychiatric disorder; (2) Pregnant women prescribed psychotropic medications, including those receiving treatment with antipsychotics, anxiolytics, and antidepressants; (3) Pregnant women who declined to cooperate with follow-up or withdrew from the study midway. For the control group, 117 normal pregnant women in the second trimester without pregnancy complications who underwent antenatal care in our hospital were included.

### 2.2 Data collection

Both online and paper questionnaires were designed to collect basic information, including name, age, height, weight, educational experience, employment situation, payment model, family income/month, gestational age, gravidity, parity, number of children, history of abnormal pregnancy, induced abortion, and spontaneous abortion.

### 2.3 Main outcome measures

Two common self-report measures, the SDS (17, 18) and the SAS (19), were adopted to evaluate the severity of depression and anxiety. As demonstrated in previous studies, both the SAS (20, 21) and the SDS (22–24) had good psychometric properties and better clinical utility and showed a remarkable ability to discriminate between normal people and those with anxiety and depression. According to many articles and the expert consensus of Chinese doctors, the evaluated individuals can be divided into normal (≦̸49), mild (50--59), moderate (60--70) and severe anxiety (≧̸70) groups on the basis of the SAS and normal (≦̸52), mild (53--62), moderate (63--72) and severe depression (≧̸72) groups on the basis of the SDS. All the participants completed the questionnaire independently on the basis of their feelings within the previous week. The questionnaires were retrieved and double-checked by researchers before analysis.

### 2.4 Statistical analysis

The Statistical Package for the Social Sciences (SPSS) 23.0 was used for data analyses. Independent samples t tests and Pearson chi-square tests were used for descriptive statistics. Pearson correlation analysis was conducted to explore the correlation between the SAS and SDS scores. A multivariate linear regression model was constructed to identify factors associated with SAS and SDS scores. A *P* value of <0.05 was considered to indicate statistical significance.

## 3 Results

As shown in **Table 1**, there were significant differences in body mass index (BMI), employment situation, payment model, family income/month, gravidity, parity, child number, history of abnormal pregnancy, and spontaneous abortion between the CI group and the control group (P<0.05). In contrast, there were no marked differences in age, gestational age, nationality, educational experience, or history of induced abortion between the two groups (*P*>0.05). The diagnosis of CI itself remains independently associated with higher anxiety scores in my model.

**Table 1:** Comparison of basic information between the two groups.

Variables	CI group (n=136)	Control group (n=117)	t	*P*
	Mean ± SD	Mean ± SD		
Age (year)	32.04 ± 4.16	32.09 ± 4.57	0.089	0.929
Gestational age (week)	21.05 ± 4.95	21.57 ± 4.76	0.850	0.396
BMI (kg/m^2^)	25.02 ± 4.06	22.64 ± 2.81	-5.339	<0.001

Variables	CI group	Control group	*χ* ^2^	*P*
	*N* (%)	*N* (%)		
Nationality				
Han nationality	125 (91.91%)	114 (97.44%)	2.691	0.101
Other nationality	11 (8.09%)	3 (2.56%)		
Educational experience				
Junior high school and below	2 (1.47%)	18 (15.38%)	0.188	0.665
Senior high school and bachelor	130 (95.59%)	77 (65.81%)		
Postgraduate and above	4 (2.94%)	22 (18.80%)		
Employment situation				
Employed	50(36.76%)	86 (73.50%)	32.688	<0.001
Unemployed	86(63.24%)	31 (26.50%)		
Payment model				
Self-paying	66 (51.47%)	25 (21.37%)	18.985	<0.001
Health insurance	70 (48.53%)	92 (78.63%)		
Family income/month (CNY)				
<10000	56 (41.18%)	20 (17.09%)	16.470	<0.001
10000 -20000	46 (33.82%)	50 (42.74%)		
20000-30000	23 (16.91%)	26 (22.22%)		
≥30000	11 (8.09%)	21 (17.95%)		
Gravidity				
1	4 (2.94%)	39 (33.33%)	61.234	<0.001
2	26 (19.12%)	40 (34.19%)		
≥3	106 (77.94%)	38 (32.48%)		
Parity				
0	88 (64.71%)	55 (47.01%)	7.260	0.007
1	44 (32.35%)	59 (50.43%)		
≥2	4 (2.94%)	3 (2.56%)		
Child numbers				
0	88 (64.71%)	60 (51.28%)	4.272	0.039
1	45 (33.09%)	55 (47.01%)		
2	3 (2.21%)	2 (1.71%)		
History of abnormal pregnancy				
Yes	128 (94.12%)	47 (40.17%)	83.320	<0.001
No	8 (5.88%)	70 (59.83%)		
History of spontaneous abortion				
Yes	120 (88.24%)	26 (22.22%)	109.610	<0.001
No	16 (11.76%)	91 (77.78%)		
History of induced abortion				
Yes	37 (27.21%)	33 (28.21%)	0.001	0.971
No	99 (72.79%)	84 (71.79%)		

As shown in **Table 2**, pregnant women in the CI group had an SAS score of 43.08 ± 9.14 and an SDS score of 49.76 ± 10.75, which were significantly higher than the SAS score of 38.01 ± 6.81 and the SDS score of 43.30 ± 9.35 in the control group (both P<0.001). The intergroup difference in SAS scores was 5.07 ± 2.33 points, and the intergroup difference in SDS scores was 6.46 ± 1.4 points. Patients with cervical insufficiency demonstrated significantly higher scores, indicating more pronounced symptoms of anxiety and depression. According to the diagnostic criteria of Zung’s self-rating scale, 19.85% and 39.71% of pregnant women in the CI group were considered to have different degrees of anxiety and depression, but only 5.98% and 17.10% of pregnant women in the control group met the same criteria (both P<0.05). In addition, up to 5.15% of patients in the CI group were diagnosed with moderate or severe anxiety, whereas no participants in the control group met the criteria. Moreover, the correlation analysis results revealed that there was a significant positive linear correlation between the SAS and SDS scores in both groups (both *P* < 0.001) (**Table 3**).

**Table 2:** Comparison of SAS scores, SDS scores, and mood disorders between the two groups.

Group	SAS score ( $\bar{x}$ ± s)	SDS score ( $\bar{x}$ ± s)	Anxiety severity (n, %)			Depression severity (n, %)		
			Normal	Mild	Moderate and above	Normal	Mild	Moderate and above
CI group (n=136)	43.08 ± 9.14	49.76 ± 10.75	109 (80.15%)	20 (14.70%)	7 (5.15%)	82 (60.29%)	38 (27.94%)	16 (11.77%)
Control group (n=117)	38.01 ± 6.81	43.30 ± 9.35	110 (94.02%)	7 (5.98%)	0 (0%)	97 (82.90%)	14 (11.97%)	6 (5.13%)
t or χ^2^	4.940	5.064	11.904			15.540		
*P*	<0.001**	<0.001**	0.002**			<0.001**		

*p < 0.05, ** *p* < 0.01.

**Table 3:** Correlation analysis of SAS and SDS scores between the two groups.

Group	r value (95% confidence interval)	*P*
CI group (n=136)	0.73 (0.64, 0.80)	<0.001**
Control group (n=117)	0.74 (0.64, 0.81)	<0.001**

*p < 0.05, ** *p* < 0.01.

To identify the factors influencing perinatal mood disorders in the second trimester, a multivariate linear regression model was constructed. A history of abnormal pregnancy and cervical insufficiency were associated with higher SAS scores (**Table 4**), whereas a history of abnormal pregnancy was also associated with higher SDS scores (**Table 5**).

**Table 4:** Multivariate linear regression model of SAS scores in the second trimester.

Variables	β (95% confidence interval)	t value	*P*
Cervical insufficiency			
No	Reference		
Yes	2.557 (0.123-4.990)	2.068	0.040*
History of abnormal pregnancy			
No	Reference		
Yes	4.663 (2.036-7.291)	3.495	0.001**
Intercept	36.135 (34.341-37.929)	39.671	<0.001**

*p < 0.05, ** *p* < 0.01.

**Table 5:** Multivariate linear regression model of SDS scores in the second trimester.

Variables	β (95% confidence interval)	t value	*P*
History of abnormal pregnancy			
No	Reference		
Yes	9.108 (6.493-11.724)	6.858	<0.001**
Intercept	40.474 (38.299-42.650)	39.643	<0.001**

*p < 0.05, ** *p* < 0.01.

## 4 Discussion

As one of the important reasons for recurrent pregnancy loss, cervical insufficiency (CI) has a tremendous effect on pregnant women, both psychologically and physically. In this study, gravidity, abnormal pregnancy, and spontaneous abortion were significantly more common in the CI group than in the control group, whereas parity and the number of children were significantly lower in the CI group. This finding was consistent with the characteristics of CI. CI is one of the main causes of second-trimester miscarriage. Most participants in this study experienced multiple abnormal pregnancies, including second-trimester miscarriages and premature deliveries, before being diagnosed with CI. Even a small number of them had unhealthy children due to premature delivery. Thus, raising their child has become their top priority. In this study, it was also confirmed that those women in the CI group had a lower ratio of employment and medical insurance coverage, as well as lower family income. Owing to the history of unexplained spontaneous abortions among these participants, especially some with a history of pregnancy loss after emergency cervical cerclage, they would be more prudent in all links in their next gestation. A large number of participants who had undergone cervical cerclage in our hospital believed that physical activity was an important reason for the poor outcome of the previous pregnancy and that staying in bed was necessary for tocolysis. Therefore, most of them chose to resign from their jobs, which also resulted in the absence of medical insurance as well as an overall decrease in their family income. In addition, long-term weight loss or staying in bed can cause weight gain, which results in an increase in BMI in the CI group (25, 26).

The results of this study indicated that the SAS and SDS scores of pregnant women with CI in the second trimester were significantly higher than those of normal pregnant women in the same stage. In addition, the incidence of anxiety and depression in pregnant women with CI in the second trimester was also much higher than that in normal pregnant women. In addition, the multivariate linear regression model revealed that a history of abnormal pregnancy was associated with higher SAS and SDS scores. For CI patients, the more abnormal pregnancies they experience, the greater the possibility of suffering from anxiety and depression. In the CI group in this study, 88.24% of pregnant women experienced spontaneous pregnancy losses, and the average gestational age at abortion was 21.14 ± 3.52 weeks. Previous adverse pregnancy outcomes can exert a profound and lasting psychological influence on women. Moreover, increasing age, declining fertility, repeated medical visits or surgical procedures, fear of losing a child again, and even a lack of empathy from husbands or families could continuously affect the mental state of women with CI. In this study, the gestational age at last miscarriage was defined as the “critical week”, which mostly occurs in the second trimester. The closer they were to the “critical week”, the greater the degree of psychological pressure they felt, not only from themselves but also from their family and society. This would increase the probability of perinatal mood disorders. Moreover, there was a strong correlation between the SAS and SDS scores, not only in the CI group but also in the control group. Owing to dramatic changes in hormone levels, body shape, interpersonal relationships, and lifestyles during pregnancy, several types of perinatal mood disorders, including but not limited to depression and anxiety, often occur simultaneously. Among the 27 participants who were diagnosed with anxiety in the CI group, 25 met the criteria for depression. Thus, healthcare professionals should attach great importance to this situation and try to perform interventions at earlier stages.

To reduce perinatal mood disorders, members of our group kept in touch with pregnant women in the CI group until six weeks after delivery and provided one-to-one professional guidance. A group chat named “Circle of Life” on the internet was established for doctors and nurses in our hospital to communicate with pregnant women and provide assistance. Online maternity schools were also founded to provide knowledge about health care, physical exercise, and diets during pregnancy. All pregnant women could receive support and communicate with doctors directly through group chats and maternity schools. All these efforts were made to alleviate their psychological pressure and avoid perinatal mood disorders, including anxiety and depression. To date, some pregnant women have completed full-term pregnancies, and none of them have experienced serious adverse events associated with perinatal mood disorders.

To reduce the incidence of perinatal mood disorders, the research team maintained contact with pregnant women in the cervical insufficiency (CI) group until 6 weeks postpartum and provided one-on-one professional guidance. Meanwhile, an online WeChat group named “Circle of Life” was established, which not only facilitates communication and assistance between medical staff of our hospital and pregnant women but also meets the demand of CI pregnant women for accessing professional guidance without leaving home. Additionally, an online maternal school was launched to provide pregnant women with knowledge on prenatal care, physical exercise, diet, and other aspects. All pregnant women can obtain support and communicate directly with doctors through the WeChat group and maternal school; these initiatives aim to alleviate their psychological pressure and prevent the occurrence of perinatal mood disorders such as anxiety and depression. Up to now, some pregnant women have successfully completed full-term pregnancies, and no serious adverse events related to perinatal mood disorders have been reported. Therefore, the “Circle of Life” support system constructed based on online platforms provides a new idea for conducting long-term and personalized health management for high-risk pregnant women. This study offers preliminary practical reference for building similar support models on a broader scale.

To the best of our knowledge, this is the first scientific attempt to analyze perinatal mood disorders in second-trimester pregnant women with CI and explore relevant risk factors. These findings are expected to provide guidance for psychological care and counseling in clinical practice. Pregnant women who undergo laparoscopic cervical cerclage return home after surgery; hence, identifying perinatal mood disorders, such as anxiety and depression, becomes difficult. Once these negative emotions cannot be detected in time, serious adverse effects may occur immediately. Therefore, a conventional evaluation of psychological conditions is recommended for pregnant women during the second trimester, especially during their “critical week”. Moreover, appropriate assessments and early intervention can improve quality of life during pregnancy and promote both maternal and infant health.

Limitations: This study adopted a cross-sectional design, which only captures the psychological status of participants at a single time point. A key limitation is the difficulty in disentangling the psychological impacts of cervical insufficiency (CI) itself from the potential effects of prior traumatic pregnancy loss, even after statistical adjustment. This study also has several additional limitations. First, data pertaining to relevant influencing factors and psychological status were collected via self-report questionnaires, which are prone to measurement bias due to subjective response tendencies. Second, given that second-trimester pregnancy loss typically occurs in the maternity wards of different hospitals, it was challenging to assess the psychological status of participants in the immediate aftermath of their most recent pregnancy loss. Finally, all participants were recruited from a single tertiary hospital in China, which may restrict the generalizability of the study’s findings. Based on these preliminary results, future multicenter studies with larger sample sizes are warranted to further investigate the prevalence and correlates of anxiety and depressive symptoms in pregnant women with CI.

## 5 Conclusions

In conclusion, this study demonstrated that women with CI suffer more psychological pressure and are more likely to develop anxiety and depression in their next pregnancy than healthy individuals are. Therefore, individualized and targeted mental care should be routinely provided for those undergoing cervical cerclage in clinical practice to prevent adverse outcomes.

## Data Availability

The original contributions presented in the study are included in the article/supplementary material. Further inquiries can be directed to the corresponding authors.
